# A Customized Bolus Produced Using a 3-Dimensional Printer for Radiotherapy

**DOI:** 10.1371/journal.pone.0110746

**Published:** 2014-10-22

**Authors:** Shin-Wook Kim, Hun-Joo Shin, Chul Seung Kay, Seok Hyun Son

**Affiliations:** Radiation Oncology, Incheon St. Mary’s Hospital, College of Medicine, The Catholic University of Korea, Seoul, Republic of Korea; NIH, United States of America

## Abstract

**Objective:**

Boluses are used in high-energy radiotherapy in order to overcome the skin sparing effect. In practice though, commonly used flat boluses fail to make a perfect contact with the irregular surface of the patient’s skin, resulting in air gaps. Hence, we fabricated a customized bolus using a 3-dimensional (3D) printer and evaluated its feasibility for radiotherapy.

**Methods:**

We designed two kinds of bolus for production on a 3D printer, one of which was the 3D printed flat bolus for the Blue water phantom and the other was a 3D printed customized bolus for the RANDO phantom. The 3D printed flat bolus was fabricated to verify its physical quality. The resulting 3D printed flat bolus was evaluated by assessing dosimetric parameters such as D_1.5 cm_, D_5 cm_, and D_10 cm_. The 3D printed customized bolus was then fabricated, and its quality and clinical feasibility were evaluated by visual inspection and by assessing dosimetric parameters such as D_max_, D_min_, D_mean_, D_90%_, and V_90%_.

**Results:**

The dosimetric parameters of the resulting 3D printed flat bolus showed that it was a useful dose escalating material, equivalent to a commercially available flat bolus. Analysis of the dosimetric parameters of the 3D printed customized bolus demonstrated that it is provided good dose escalation and good contact with the irregular surface of the RANDO phantom.

**Conclusions:**

A customized bolus produced using a 3D printer could potentially replace commercially available flat boluses.

## Introduction

Since the discovery of X-rays over one hundred years ago, radiotherapy has been used for the treatment of tumors. In order to deliver a sufficient radiation dose to the tumor, adequate types of radiation are selected depending on the tumor location. Conventionally, high-energy photon is used to treat deeply located lesions and electron is used for the treatment of superficial lesions such as skin cancer.

The International Commission on Radiation Units and Measurements Report 62 recommends that the target volume be encompassed within the area that receives at least 95% of the prescribed dose when radiotherapy is administered [1]. However, a sufficient dose cannot be delivered to the surface due to the skin sparing effect of high-energy radiation beams. To avoid this limitation, several types of commercially available boluses are often used [2]. These bolus materials should be nearly tissue equivalent and allow a sufficient surface dose enhancement.

Despite the advent of commercial boluses and the modernization of clinical equipment, uncertainties in the preparation and utilization of a bolus remain [3]. In practice, most commonly used commercial flat boluses cannot form perfect contact with the irregular surface of the patient’s skin, particularly the nose, ear, and scalp, and the resulting air gap affects the second skin sparing effect and reduces both the maximum and surface dose [4]–[8]. Even more problematic though, is that the depth of the air gap cannot be anticipated and thus accounted for in the treatment planning step, leading to a discrepancy between the planned and delivered dose. Thus, commercial flat boluses need to be used with great care, especially when the skin has a particularly irregular shape.

Recently, there have been significant advances in 3-dimensional (3D) printer technology, and attempts have been made to utilize them in medicine [9], [10]. In this study, we fabricated a customized bolus using a 3D printer and assessed whether it could overcome the disadvantages of currently used commercial flat boluses.

## Materials and Methods

### Bolus fabrication using a 3D printer

**Figure 1:**
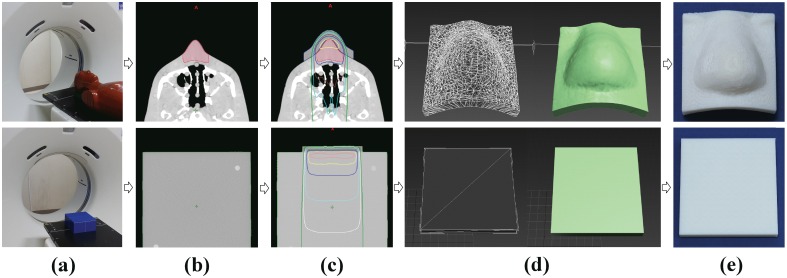
Schematic illustration of the procedures for 3-dimensional (3D) printing of a bolus. (a) Obtaining computed tomography images for the RANDO phantom (top) and the Blue water phantom (bottom). (b) Delineation of a body contour and a target volume using the treatment planning system. (c) The optimized bolus structure designed to sufficiently cover the target volume. (d) 3D rendering of the bolus structure. (e) The fabricated 3D printed customized bolus (top) and flat bolus (bottom).

For this study, we used the Blue water phantom (Standard Imaging, Middleton, WI) as a homogenous phantom and the RANDO phantom (Radiology Support Devices, Long Beach, CA) as an anthropomorphic phantom. Computed tomography (CT) images of the Blue water phantom and the RANDO phantom were obtained using a LightSpeed RT 16 CT scanner (GE Medical Systems, Waukesha, WI) in the general digital imaging and communications in medicine (DICOM) format (Figure 1a). The CT scanning conditions were as follows: slice thickness, 1.25 mm; peak voltage, 120 kVp; current, 440 mA (Auto); noise index, 7.35; pitch, 0.938; and display field of view, 30 cm. Eclipse ver. 8.9 (Varian Medical Systems, Palo Alto, CA) with the Anisotropic Analytical Algorithm was used as a treatment planning system (TPS). The conditions for body contouring were −500 Hounsfield units (HU) without the use of the smoothing option.

Based on these CT images, we fabricated a 3D printed flat bolus on the Blue water phantom to verify its physical quality. To fully cover the 10×10 cm open field, the 3D printed flat bolus was sized at 11×11 cm (Figure 1c, bottom), with a thickness of 1 cm in order to escalate the dose at the buildup region of a 6 megavoltage (MV) photon beam. The RANDO phantom was used to fabricate a 3D printed customized bolus, for which a virtual target volume was delineated below the surface of the phantom, and the size of the target volume was 4×4 cm around its nose (Figure 1b, top). To completely cover the 5 cm×5 cm open field, the bolus was sized at 7×7 cm (Figure 1c, top), and the treatment plan was designed to cover 90% of the target volume with 90% of the prescribed dose. In order to meet this condition, the printed bolus was made 1 cm thick.

Although the bolus was designed using Eclipse, the 3D-rendered structure cannot be directly converted into the stereolithography (STL) format. Therefore, we performed the following additional steps. OsiriX MD ver. 2.8.x (OsiriX, Geneva, Switzerland) was used for 3D rendering of the designed bolus structure in DICOM-RT format. In order to convert the file into the STL format, 3Ds Max 2013 (Autodesk, San Rafael, CA) was used (Figure 1d). Insight ver. 9.1 (Stratasys, Eden Prairie, MN) was used to print out the STL file of this designed bolus on a Fortus 400 mc 3D printer (Stratasys, Eden Prairie, MN). The Fortus 400 mc is a fused deposition modeling technique 3D printer, and we used a 0.254 mm layer deposition (variation: ±0.127 mm per mm) for printing. The time required for 3D printing of the flat bolus and customized bolus was 3 and 4.5 hours, respectively. The bolus material was ABS-M30 (Stratasys, Eden Prairie, MN), a form of acrylonitrile butadiene styrene that is commonly used by these devices. This material has a density of 1.04 g/cm^3^ and −123.6±18.2 HU at 120 kVp. A schematic representation of this process is shown in Figure 1.

### Evaluation of the 3D printed bolus

In order to evaluate the 3D printed bolus, treatment plans were generated for the Blue water phantom without a bolus, with a superflab bolus, and with the 3D printed flat bolus. All of the plans were set at 200 monitor units (MU) with a single 6 MV photon beam. The parameters of the treatment plan were a 0 degree gantry angle, a 6 MV photon beam, a 10×10 cm, open field, a 100 cm surface to source distance (SSD), and a 0.25 cm calculation grid. For absolute dose measurement, a farmer type ionization chamber (Exradin A19 ionchamber, Standard Imaging, Middleton, WI) and SuperMAX electrometer (Standard Imaging, Middleton, WI) were used. For dose profile measurement, Gafchromic EBT2 film (International Specialty Products, Wayne, NJ) was used. An Epson Perfection V700 Photo Scanner (Epson, Long Beach, CA) was used to determine the optical density of the films and ImageJ ver. 1.47 v (National Institutes of Health, Bethesda, MD) was used for the film analysis. The measurement parameters were the same as those of each plan. All plans were compared with the percent depth dose (PDD) measured from the film and TPS, and depth doses (D_d_) measured from the ionization chamber at the central axis. The dosimetric parameters are defined below.

Treatment plans were then generated for the RANDO phantom without a bolus and with the 3D printed customized bolus. The parameters of the treatment plan were a 0 degree gantry angle, a 6 MV photon beam, a 5×5 cm, open field, a 100 cm SSD, and a 0.25 cm calculation grid. The plan with the 3D printed customized bolus was set so that 90% of the prescribed dose was delivered to 90% of the target volume, and the plan without a bolus and the plan with the 3D printed customized bolus were normalized to the same maximum dose of the target volume. Both plans were compared in terms of the percent depth dose (PDD) at the central axis and the dose volume histogram (DVH) of the target volume. The D_max_, D_min_, D_mean_, D_90%_, and V_90%_ of the treatment plans were compared. These dosimetric parameters are defined below:

1. d_max_: depth of maximum dose from the surface of the phantom
2. D_d_: absorbed dose at d cm beneath the surface of the phantom
3. D_max_: maximum dose of the target volume
4. D_min_: minimum dose of the target volume
5. D_mean_: mean dose of the target volume
6. D_90%_: the dose that covers 90% of the target volume
7. V_90%_: the target volume that receives over the 90% of the prescribed dose

## Results

### The 3D printed flat bolus on the blue water phantom

**Figure 2:**
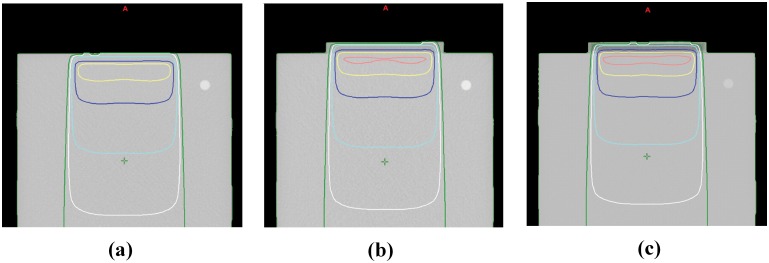
Dose distributions of the three treatment plans from the Blue water phantom study. (a) Plan without a bolus, (b) plan with the superflab bolus, and (c) plan with the 3-dimensional printed flat bolus. Pink line, 105% isodose contour; yellow line, 100% isodose contour; blue line, 90% isodose contour; cyan line, 70% isodose contour; white line, 50% isodose contour; dark green, 30% isodose contour.

The 3D printed flat bolus was successfully fabricated using the 3D printer (Figure 1e, bottom), and was a good fit against the surface of the Blue water phantom with no air gap between the bolus and the phantom. The dose distribution of the plan without a bolus revealed that the prescribed dose could not be fully delivered to the surface of the Blue water phantom (Figure 2a). The d_max_ of this plan was calculated with a TPS of 1.48 cm, whereas the plan with the 3D printed flat bolus produced a d_max_ of 0.63 cm. Thus, the d_max_ of the plan with the 3D printed flat bolus was shifted 0.85 cm in depth towards the surface of the Blue water phantom, suggesting that the 3D printed flat bolus was also a useful dose escalating material in radiotherapy. Moreover, the dose distributions of the plans with the superflab and the 3D printed flat bolus were similar, as expected (Figure 2b, c). The differences between the calculated dose by the TPS and the measured dose from the ionization chamber at a depth of 1.5 cm, 5 cm, and 10 cm beneath the surface of the phantom are shown in Table 1.

**Table 1:** Comparison of the dosimetric parameters of each plan from the Blue water phantom study.

Parameters	D_1.5 cm_			D_5 cm_			D_10 cm_		
	Cal(Gy)	Mea(Gy)	Diff(%)	Cal(Gy)	Mea(Gy)	Diff(%)	Cal(Gy)	Mea(Gy)	Diff(%)
Without a bolus	2.00	1.99	0.50	1.72	1.71	0.58	1.32	1.31	0.76
With the superflab bolus	1.98	1.98	0.00	1.67	1.66	0.60	1.27	1.26	0.79
With the 3D printed flat bolus	1.99	1.99	0.00	1.68	1.67	0.60	1.28	1.27	0.78

D_1.5 cm_: absorbed dose at 1.5 cm depth beneath the surface of the phantom; D_5 cm_: absorbed dose at 5 cm depth beneath the surface of the phantom; D_10 cm_: absorbed dose at 10 cm depth beneath the surface of the phantom; Cal: calculated dose from the treatment planning system; Mea: measured dose from an ionization chamber; Diff: Differences between the calculated dose and the measured dose.

**Figure 3:**
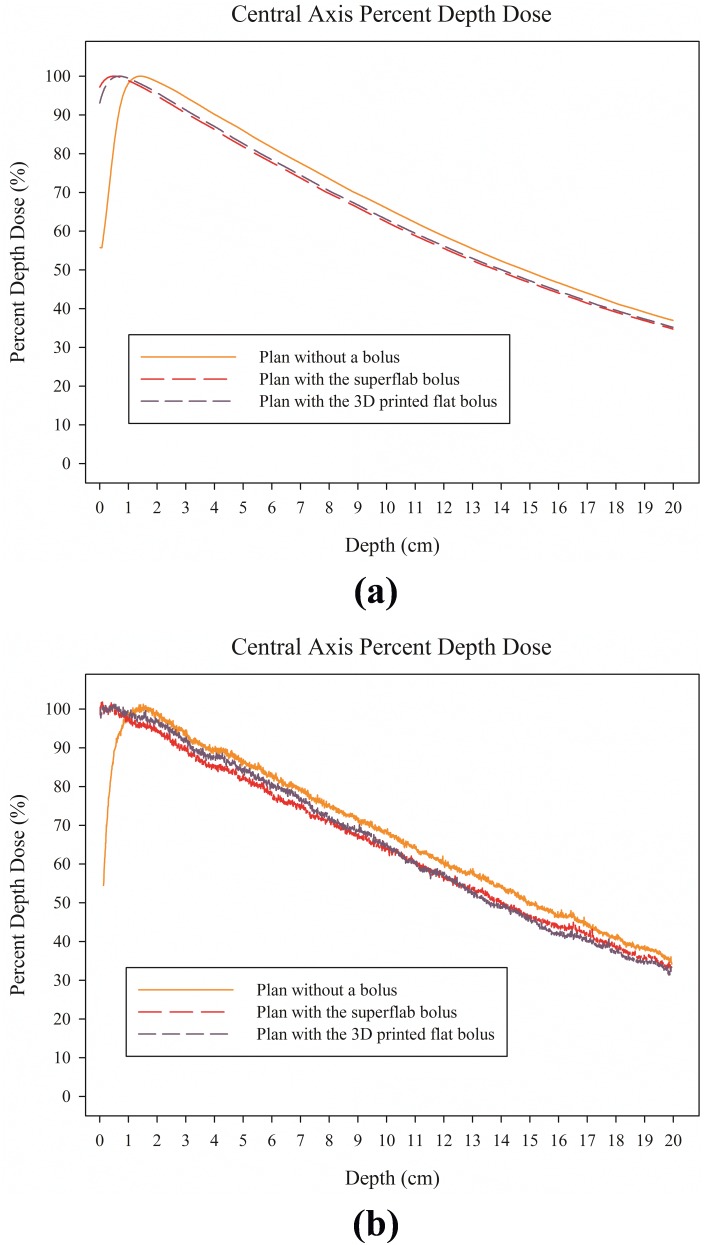
Percent depth dose (PDD) at the central axis in the Blue water phantom study. (a) PDD calculated from the treatment planning system and (b) PDD measured from the film.

The differences between the calculated and measured doses were less than 1%, indicating that the dose distribution can be calculated to a very high degree of accuracy when the 3D printed bolus was applied. The PDD from the TPS and film dosimetry are shown in Figure 3, and the shapes of the plots of the calculated PDD from the TPS are similar to those for the measured PDD from the film.

### The 3D printed customized bolus on the RANDO phantom

**Figure 4:**
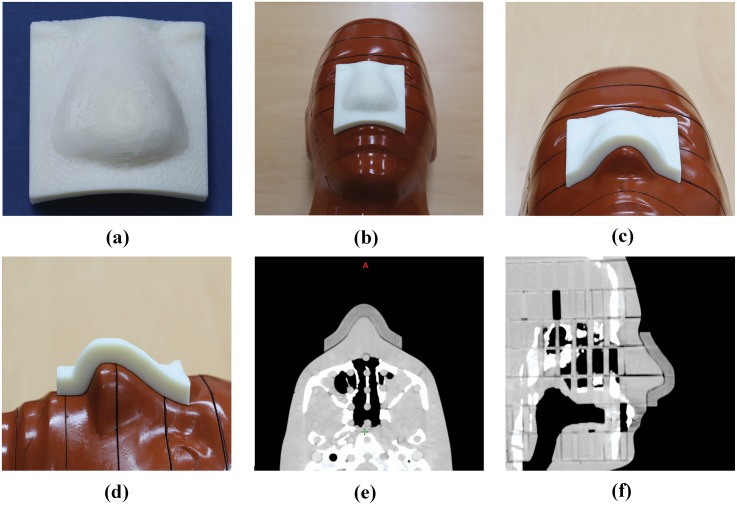
The 3-dimensional (3D) printed customized bolus. (a) The 3D printed customized bolus, (b) the 3D printed customized bolus on the surface of the RANDO phantom, (c, d) cross sectional view of the 3D printed customized bolus, and (e, f) axial and sagittal CT images of the 3D printed customized bolus on the RANDO phantom.

Figure 4a shows the 3D printed customized bolus produced using the 3D printer, and Figure 4b shows it positioned on the surface of the RANDO phantom. On visual inspection, the 3D printed customized bolus was found to fit well against the surface of the RANDO phantom, and this was verified in cross section (Figure 4c, d) and by CT imaging (Figure 4e, f).

**Figure 5:**
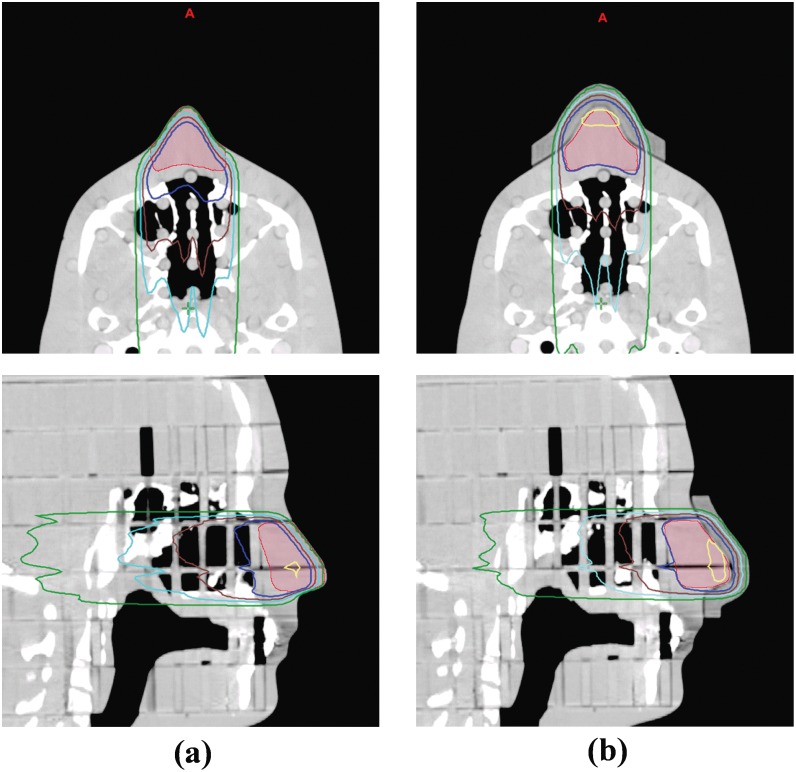
Dose distributions of the two treatment plans from the RANDO phantom study. (a) Plan without a bolus, (b) plan with the 3D printed customized bolus. Yellow line, 100% isodose contour; blue line, 90% isodose contour; brown line, 80% isodose contour; cyan line, 70% isodose contour; dark green line, 50% isodose contour.

**Figure 6:**
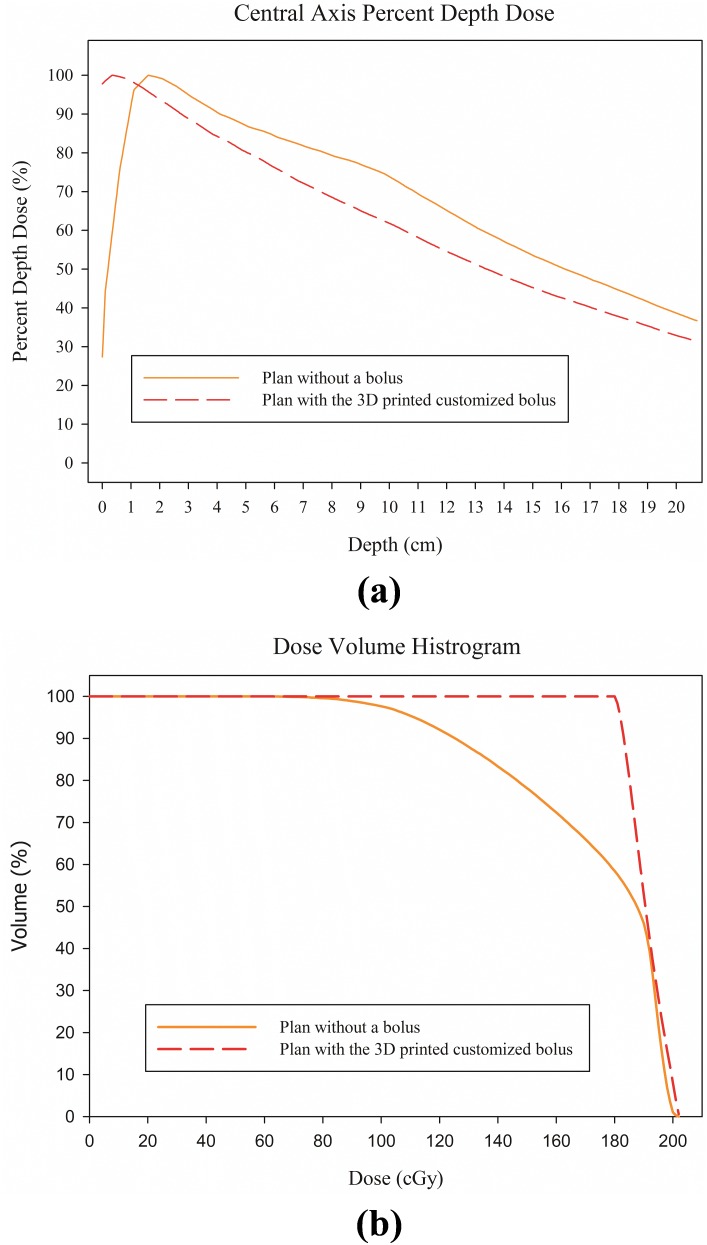
Percent depth dose (PDD) at the central axis and the dose volume histogram (DVH) of each plan from the RANDO phantom study. (a) PDD, (b) DVH.

For the RANDO phantom study, the dose distributions of the plans without a bolus and with the 3D printed customized bolus on the RANDO phantom are shown in Figure 5, indicating that the 3D printed customized bolus is a good buildup material. For the plan without a bolus, the D_max_, D_min_, D_mean_, D_90%_, and V_90%_ of the target volume were 101.3%, 25.4%, 86.4%, 62.7%, and 53.5%, respectively. This suggests that the plan without a bolus cannot fully deliver the prescribed dose to the target volume. However, when the 3D printed customized bolus was added, the D_max_, D_min_, D_mean_, D_90%_, and V_90%_ of the target volume were 101.3%, 90.0%, 95.5%, 91.6%, and 100.0%, respectively, indicating effective dose coverage. Each dosimetric parameter is shown in Table 2, and the PDD and DVH of both plans are illustrated in Figure 6. Both the PDD and DVH show sufficient dose escalation in the target volume with the 3D printed customized bolus.

**Table 2:** Comparison of the dosimetric parameters of each plan from the RANDO phantom study.

Parameters	D_max_(%)	D_min_(%)	D_mean_(%)	D_90%_(%)	V_90%_(%)
Without a bolus	101.3	25.4	86.4	62.7	53.5
With the 3D printed customized bolus	101.3	90.0	95.5	91.6	100.0

D_max_: maximum dose of the target volume; D_min_: minimum dose of the target volume; D_mean_: mean dose of the target volume; D_90%_: the dose that covers 90% of the target volume; V_90%_: the target volume that received over 90% of the prescribed dose.

## Discussion

The aim of radiotherapy is to deliver a sufficient radiation dose to a defined tumor, whilst minimizing the dose to the surrounding healthy tissue. High-energy photon is widely used in modern radiotherapy. However, it exhibits a skin sparing effect derived from the buildup region. This is regarded as advantageous when the tumors are in a deep location as damage to the skin and its resulting complications are avoided. On the other hand, if the tumors are superficial, the skin sparing effect reduces the tumor dose and could result in treatment failure. For the treatment of tumors on or near the skin, the skin sparing effect needs to be overcome in order to reduce the risk of recurrence. In order to achieve this, a bolus is placed on the patients’ skin. However, commonly used flat bolus materials cannot make perfect contact with this irregular surface, leaving an unwanted air gap between the two. Butson *et al.* reported that approximately 6–10% of the surface dose, depending on the field size and angle of incidence, was reduced when using a 6 MV photon beam in the presence of a 10-mm air gap [4]. Khan *et al.* have also studied the dose perturbations of a 6 MV photon beam. They found that the surface dose is significantly affected by air gaps greater than 5 mm [5]. In the case of electron beams, several studies have investigated the dose reduction resulting from air gaps, with similar results to those obtained with photon beams [7], [8]. However, an air gap might be unavoidable in the routine daily patients’ setup. Even more problematic is that the depth of the air gap cannot be anticipated and thus calculated at the treatment planning step. As a result, there might be a discrepancy between the planned and delivered doses.

In this study, we fabricated a 3D printed flat bolus and evaluated its properties as a bolus material. As shown in Figure 2c and Table 1, the 3D printed flat bolus can provide effective dose coverage in the buildup region. The d_max_ of the plan with the superflab and 3D printed flat bolus were shifted toward the surface of the Blue water phantom by as much as 0.91 cm and 0.85 cm, respectively. There were slight differences between the dosimetric results obtained using these boluses because the 3D printed flat bolus is not identical to the superflab bolus with respect to its HU value and density. At 120 kVp, the HU of the 3D printer bolus was −123.6±18.2 HU compared to was −33.04±7.6 HU for the superflab bolus. In addition, the commercially available flat boluses and the 3D printed flat bolus also do not have completely homogeneous HU values, potentially giving rise to variation in the measured doses at the central axis.

We fabricated a customized 3D bolus using a 3D printer and evaluated its feasibility in clinical practice by comparing its performance with treatment plan without a bolus. As shown in Figure 4, the 3D printed bolus is a good fit against the irregular surface of the RANDO phantom, and the resulting dosimetric parameters of the plan without a bolus and with the 3D printed customized bolus on the surface of the RANDO phantom indicated that the 3D printed customized bolus is a good buildup material. Furthermore, the treatment plan with the 3D printed customized bolus could be clinically effective, help to overcome the problem of variable air gaps, and improve reproducibility of daily setup conditions on irregular surfaces compared to commercial flat boluses.

## Conclusions

The customized bolus produced by a 3D printer could potentially replace and improve upon commercially available flat boluses. The 3D printed boluses can increase the reproducibility of daily setup and help overcome some of the disadvantages of currently used commercially available flat boluses. The 3D printed bolus could therefore also increase the efficacy of the radiotherapy.
